# (Non)-Exertional Variables of Cardiopulmonary Exercise Testing in Heart Failure with and Without Cardiac Amyloidosis

**DOI:** 10.1007/s11897-024-00661-1

**Published:** 2024-04-18

**Authors:** Simon Wernhart, Lars Michel, Alexander Carpinteiro, Peter Luedike, Tienush Rassaf

**Affiliations:** 1 Department of Cardiology and Vascular Medicine, West German Heart and Vascular Center, University Hospital Essen, University Duisburg-Essen, Hufelandstrasse 55, 45147 Essen, Germany; 2 Clinic for Hematology and Stem Cell Transplantation, University Hospital Essen, University Duisburg-Essen, Hufelandstrasse 55, 45147 Essen, Germany

**Keywords:** CPET, Cardiac amyloidosis, Chronic heart failure, Non-exertional variables, Phenotyping

## Abstract

**Purpose of Review:**

Cardiac amyloidosis (CA) constitutes an important etiology of heart failure with preserved ejection fraction (HFpEF) or heart failure with mildly reduced ejection fraction (HFmrEF). Since patients with CA show early exhaustion, we aimed to investigate whether non-exertional variables of cardiopulmonary exercise testing (CPET) provide additional information in comparison to traditional peak oxygen consumption (VO_2peak_).

**Recent Findings:**

We retrospectively investigated CPET variables of patients with HFpEF and HFmrEF with (*n* = 21) and without (*n* = 21, HF) CA at comparable age and ejection fraction. Exertional and non-exertional CPET variables as well as laboratory and echocardiographic markers were analyzed. The primary outcome was the difference in CPET variables between groups. The secondary outcome was rehospitalization in patients with CA during a follow-up of 24 months. Correlations between CPET, NTproBNP, and echocardiographic variables were calculated to detect patterns of discrimination between the groups. HF patients with CA were inferior to controls in most exertional and non-exertional CPET variables. Patients with CA were hospitalized more often (*p* = 0.002), and rehospitalization was associated with VE/VCO_2_ (*p* = 0.019), peak oxygen pulse (*p* = 0.042), the oxygen equivalent at the first ventilatory threshold (*p* = 0.003), circulatory (*p* = 0.024), and ventilatory power (*p* < .001), but not VO_2peak_ (*p* = 0.127). Higher performance was correlated with lower E/e’ and NTproBNP as well as higher resting heart rate and stroke volume in CA.

**Summary:**

Patients with CA displayed worse non-exertional CPET performance compared to non-CA HF patients, which was associated with rehospitalization. Differences between correlations of resting echocardiography and CPET variables between groups emphasize different properties of exercise physiology despite comparable ejection fraction.

## Introduction

Cardiac amyloidosis (CA), either triggered by light chain (AL) or transthyretin (TTR) deposition in the heart, results in a restrictive relaxation pattern and heart failure (HF) with reduced prognosis [1]. Exercise limitations can be directly measured by cardiopulmonary exercise testing (CPET), which presents prognostic information in patients with HF [2•]. Patients with CA may display chronotropic insufficiency, reduced exercise capacity expressed by peak oxygen consumption (VO_2peak_), and inefficient ventilatory response measured by increased minute ventilation to carbon dioxide production (VE/VCO_2_) [3•, 4]. As these variables have been extensively studied in chronic HF across all categories, compound variables such as circulatory power (CP), calculated at peak exercise by the product of VO_2peak_ x peak systolic blood pressure [5], and ventilatory power (VP), measured as the ratio of peak systolic blood pressure and VE/VCO_2_ [6], have been shown to be of prognostic value in advanced HF. The role of VP has scarcely been studied in a CA population. Submaximal variables such as oxygen uptake efficiency slope (OUES) or the oxygen equivalent at the first ventilatory threshold (EqO_2_ at VT1) have not been investigated in CA patients.

Patients with CA often display preserved or mildly reduced ejection fractions despite severe limitations in exercise capacity and prognosis [7]. However, ejection fraction is insufficient to discriminate etiologies of HF, including patients with HFpEF and HFmrEF. Current position papers recommend CPET in the determination of training corridors for HF and CA [7–9], but different patterns of exercise limitations may further help to discriminate CA from other aetiologies of HF while resting echocardiography still does not raise suspicion for CA. As CA patients often suffer from severely compromised exercise capacity, exertional variables, such as VO_2peak_ which require maximal metabolic exertion, may not accurately depict the true impact of exercise intolerance. We thus aimed to compare exertional and non-exertional CPET variables between patients with CA and other HF aetiologies at a comparable age and ejection fraction to better understand systemic exercise limitations in CA as opposed to HF patients. We also aimed to investigate associations between CPET and resting echocardiographic variables to delineate potential differences of exercise response in HF and CA patients.

## Methods

### Setting and Participants

We retrospectively analyzed clinically stable but symptomatic patients diagnosed with HFpEF (≥ 50%) or HFmrEF (41–49%) according to current guidelines [10] who underwent CPET as a part of their regular outpatient visit. This population of patients is highly relevant for clinical practice since half of all HF patients display preserved or only mildly reduced ejection fraction and may benefit from different medical treatment compared to HF with reduced ejection fraction [11]. CPET may be an important tool in clinical practice to guide medical therapy, as it can identify reduced chronotropic competence, stroke volume increase, and peripheral oxygen extraction as major determinants of dyspnoea despite relatively preserved ejection fraction at rest. Indications for CPET were provided by the supervising physician due to the suspicion of clinical deterioration (patient’s reporting of loss of one NYHA class compared to the prior visit) to assess exercise capacity. Patients had to be above 18 years of age and reported dyspnoea NYHA II or III during exercise. Twenty-four months after the start of our CPET database, a preliminary analysis was performed, and patients with HpEF and HFmrEF were categorized into a group of cardiac amyloidosis (CA) and HF controls with cardiomyopathy of other aetiologies than CA. CA was diagnosed following the algorithm of current recommendations [7]. Patients with reduced ejection fraction (≤ 40%), valvular heart disease, younger age (< 18 years), an indication for coronary angiography, or NYHA I or IV were excluded. The study protocol conforms to the ethical guidelines of the 1975 Declaration of Helsinki and was approved by the local ethics committee of the Faculty of Medicine of the University Duisburg-Essen, Germany (22–10562-BO).

### Cardiopulmonary Exercise Protocol

We performed a ramp protocol on a bicycle ergometer (eBike II, GE Healthcare, Chicago, IL, USA) with an exercise duration of 8–12 min, starting at a workload of 10 W with an increment of 10 W/min and a pedalling rate of 60 rounds per minute. A metabolic cart interface (Vyntus™ CPX Metabolic Cart, Vyaire Medical, Hoechberg, Germany) was used to measure respiratory gas exchange. Ventilatory thresholds and data interpretations were performed by an exercise physiologist with licensed software (SentrySuite™ Software Solution, Vyaire™ Medical, Hoechberg, Germany). Percentage of age-predicted VO_2peak_ (% of pred VO_2peak_) was calculated using the Wasserman-Hansen equation [12]; exercise oscillatory ventilation (EOV) was determined according to a commonly used algorithm applied by the software [13]. O_2_ pulse was related to body weight and multiplied by 100 for better readability [14]; plateauing of the O_2_ pulse was visually assessed by a flattening of the curve. The oxygen equivalent at the first ventilatory threshold (EqO_2_ at VT1) [15, 16] and oxygen uptake efficiency slope (OUES), the relation of oxygen uptake, and the logarithmic minute ventilation [17] were assessed as previously recommended. Increase of PETCO_2_ > 3 mmHg during exercise was deemed acceptable as a surrogate for sufficient alveolar perfusion during exercise [15]. Dead space ventilation (*V*_D_/*V*_T_) was estimated from end tidal CO_2_ (PETCO_2_), capillary CO_2_ as an approximation of arterial CO_2_ partial pressure (paCO_2_), tidal volume (*V*_T_), and tidal volume of the breathing valve (*V*_BV_, 0.075 L) following the formula:

$$\frac{{V}_{D}}{{V}_{T}}=\left[\frac{paC{O}_{2}-PETC{O}_{2}}{paC{O}_{2}}\right]-{V}_{BV}/({V}_{T}-{V}_{BV})$$

Heart rate reserve (ΔHR) was calculated as the difference between resting and peak heart rate. Systolic and diastolic blood pressure were measured every minute with a standard upper arm cuff at peak exercise and each minute during the 3-min active recovery period (cycling at 25 W). VE/VCO_2_ slope was calculated using linear regression analysis between the start and end of the test [18]. CP was calculated at peak exercise by the product of VO_2peak_ x peak systolic blood pressure, while VP was measured as the ratio of peak systolic blood pressure and VE/VCO_2_. Exercise tests were performed until maximal subjective exertion (a BORG scale ≥ 18 points). Criteria for premature exercise termination were defined according to established guidelines [15]. Patients were advised to take their morning medication on the day of exercise testing to mirror patients’ daily routine.

### Co-Variable Assessment

Transthoracic echocardiography was performed within 48 h of CPET by an experienced cardiologist according to established recommendations [19]. Severity of relevant (at least grade 2) valve dysfunction was assessed qualitatively and semi-quantitatively according to current recommendations [20]. Cardiac output (CO) at rest was calculated during echocardiography from resting heart rate (HR_rest_) and stroke volume (SV) determined by left ventricular outflow tract diameter times the velocity time integral. Tricuspid annular plane systolic excursion (TAPSE) was used as a surrogate for right ventricular function. Laboratory values were taken on the day of reporting to the clinic.

To analyze systemic exercise limitations in CA patients, we compared exertional and non-exertional CPET variables in CA patients with a HF control group of different etiologies but comparable age and ejection fraction as a primary outcome. We also aimed to assess the prognostic utility of exertional and non-exertional CPET variables to assess rehospitalization in patients with CA over a follow-up period of 24 months as a secondary outcome. Rehospitalization rates were monitored from the local hospital database, and medical history was taken during every outpatient visit. Correlation coefficients between CPET and echocardiographic variables as well as the biomarker NTproBNP were calculated as an attempt to delineate phenotypes with more severe exercise limitations and to identify potential patterns for earlier diagnosis of CA.

### Statistical Methods

SPSS (IBM Corp. Released 2016. IBM SPSS Statistics for Windows, Version 24.0. Armonk, NY: IBM Corp.) and the R-program [21] were used for data analysis and graphical depiction of results. Baseline characteristics were calculated by descriptive statistics, and normal distribution was tested with the Shapiro–Wilk test. Non-normally distributed values were presented in quartiles. Effects of CPET variables on groups were evaluated by the exact Fisher test (nominal scale). Nonparametric *U*-test was applied to assess differences in groups in quantitative, ratio-scaled measurements. As a level of significance, *α* was set at 0.05. Correlations between CPET variables, biomarkers, and echocardiographic variables were analyzed using Spearman’s correlation coefficients.

## Results

### Baseline Characteristics and Group Differences

Patients were of comparable age, but HF control patients were more obese (Table 1), hypertensive (*n* = 15 vs. *n* = 4, *p* < 0.001), and contained more smokers (*n* = 8 vs. *n* = 2, *p* = 0.030), but there was no difference in coronary artery disease (*n* = 4, *n* = 2, *p* = 0.390). Similarly, to patients with CA, the etiology of HF in the control group was primarily non-ischemic (*n* = 17) and consisted of patients with non-dilated cardiomyopathy (*n* = 15), while two patients suffered from dilated cardiomyopathy. CA mainly consisted of patients with wild type (*n* = 12) and hereditary (*n* = 6) ATTR amyloidosis (*n* = 18), and three patients with AL-amyloidosis (*n* = 3 in Mayo class IIIa) with diagnosed cardiac involvement who had received combination therapy with daratumumab, cyclophosphamide, bortezomib, and dexamethasone at least 2 weeks prior to CPET. The group of HF patients was characterized by hypertensive (*n* = 8), dilated (*n* = 2), or ischemic (*n* = 13) cardiomyopathy; no patients with known hypertrophic cardiomyopathy were included.

**Table 1:** Comparison of baseline characteristics in patients with cardiac amyloidosis (CA) and heart failure controls (HF)

Variables	Min	Q1	Q2	Q3	Max	*p*-value
**Age (years)**						*p* = 0.734
CA (*n* = 21)	44.0	56.0	58.0	61.0	81.0	
HFpEF (*n* = 21)	53.0	58.0	61.0	63.0	65.0	
**BMI (kg/m**^**2**^**)**						*p* = 0.003*
CA (*n* = 21)	16.0	23.0	25.0	27.0	36.0	
HFpEF (*n* = 21)	20.0	26.0	29.0	33.0	42.0	
**NTproBNP (pg/ml)**						*p* < 0.001*
CA (*n* = 21)	484.0	1180.0	3943.0	12,602.0	30,437.0	
HFpEF (*n* = 21)	31.0	54.0	89.0	203.0	1222.0	
**Hemoglobin (g/dl)**						*p* = 0.056
CA (*n* = 21)	9.1	13.0	14.0	14.0	17.0	
HFpEF (*n* = 21)	12.9	13.8	14.6	15.2	15.9	
**eGFR (ml/min)**						*p* = 0.012*
CA (*n* = 21)	31.8	58.0	63.0	72.0	81.0	
HFpEF (*n* = 21)	59.0	66.0	72.0	76.0	92.0	
**Thrombocytes (/nl)**						*p* = 0.869
CA (*n* = 21)	60.0	159.0	220.0	278.0	383.0	
HFpEF (*n* = 21)	88.0	190.0	235.0	245.0	332.0	
**HR**_**rest**_** (/min)**						*p* < 0.001*
CA (*n* = 21)	45.0	54.0	56.0	63.0	69.0	
HFpEF (*n* = 21)	54.0	65.0	67.0	75.0	93.0	
**SV (ml)**						*p* < 0.001*
CA (*n* = 21)	40.0	45.0	48.0	51.0	65.0	
HFpEF (*n* = 21)	51.0	61.0	65.0	69.0	86.0	
**CO (ml/min)**						*p* < 0.001*
CA (*n* = 21)	2070.0	2520.0	2646.0	2898.0	4095.0	
HFpEF (*n* = 21)	3240.0	3953.0	4680.0	5250.0	5925.0	
**LVEF (%)**						*p* = 0.084
CA (*n* = 21)	40.0	50.0	54.0	57.0	67.0	
HFpEF (*n* = 21)	43.0	54.0	59.0	62.0	70.0	
**LVMI (g/m**^**2**^**)**						*p* < 0.001*
CA (*n* = 21)	85.6	133.0	172.0	201.0	482.0	
HFpEF (*n* = 21)	64.3	83.8	96.4	106.9	133.6	
**LAVI (ml/m**^**2**^**)**						*p* < 0.001*
CA (*n* = 21)	24.2	37.0	43.0	48.0	71.0	
HFpEF (*n* = 21)	13.8	21.7	24.6	32.1	69.5	
**TAPSE (mm)**						*p* < 0.001*
CA (*n* = 21)	13.0	18.0	19.0	21.0	25.0	
HFpEF (*n* = 21)	18.0	20.0	26.0	27.0	32.0	
**E/e’**						*p* < 0.001*
CA (*n* = 21)	12.3	14.0	16.0	18.0	22.0	
HFpEF (*n* = 21)	8.2	10.2	11.2	12.3	16.5	

Note: Differences of baseline characteristics are calculated with the exact Fisher and Mann–Whitney *U*-tests. Quartiles (*Q*) are depicted for each group. *Averaged E/e’* Mean of lateral and medial E/e’ were used to assess diastolic function during resting echocardiography. *CO* cardiac output; this was calculated by echocardiographic determination of stroke volume times resting heart rate during echocardiography. *HR*_rest_ resting heart rate during echocardiography, *MRA* mineralocorticoid receptor antagonist, *eGFR* estimated glomerular filtration rate, *LAVI* left atrial volume index, *LVEF* left ventricular ejection fraction, *LVMI* left ventricular mass index, *NTproBNP* N-terminal prohormone of brain natriuretic peptide, *SV* stroke volume was calculated by echocardiographic measurement of left ventricular outflow tract diameter and the velocity time integral, *TAPSE* tricuspid annular plane systolic excursion

Significance was denoted with an asterisk

The groups did not differ in sex (females: in HF *n* = 8, in CA *n* = 3, *p* = 0.083), diabetic status (*n* = 4 each), atrial fibrillation (HF *n* = 5, CA: *n* = 8, *p* = 0.329), and coronary artery disease (HF *n* = 4, CA: *n* = 2, *p* = 0.390), while there were more hypertensive (*n* = 15 vs. *n* = 4; *p* < 0.001) and smoking (*n* = 8 vs. *n* = 2, *p* = 0.030) HF patients. There were more rehospitalizations due to HF in CA (*n* = 11 vs. *n* = 2, *p* = 0.002); only two patients in the CA group died during the follow-up period undergoing palliative care. Beta-blocker administration (*p* = 0.367), sodium-glucose co-transporter inhibitors (*p* = 0.122), and angiotensin receptor blockers (*p* = 0.062) were not different between the groups, while mineralocorticoid receptor antagonists were more often prescribed in CA (*p* = 0.013). There were considerable differences in NTproBNP between groups (*p* < 0.001), as well as in the echocardiographic variables of left ventricular mass index, left atrial volume, and right ventricular (all *p* < 0.001), but not in left ventricular function (*p* = 0.084), or valvular disease (HF *n* = 2, CA: *n* = 5, *p* = 0.224). SV, Hf_rest_, and CO were lower, while average E/e’ was higher in CA patients (all *p* < 0.001, Table 1).

**Fig. 1:**
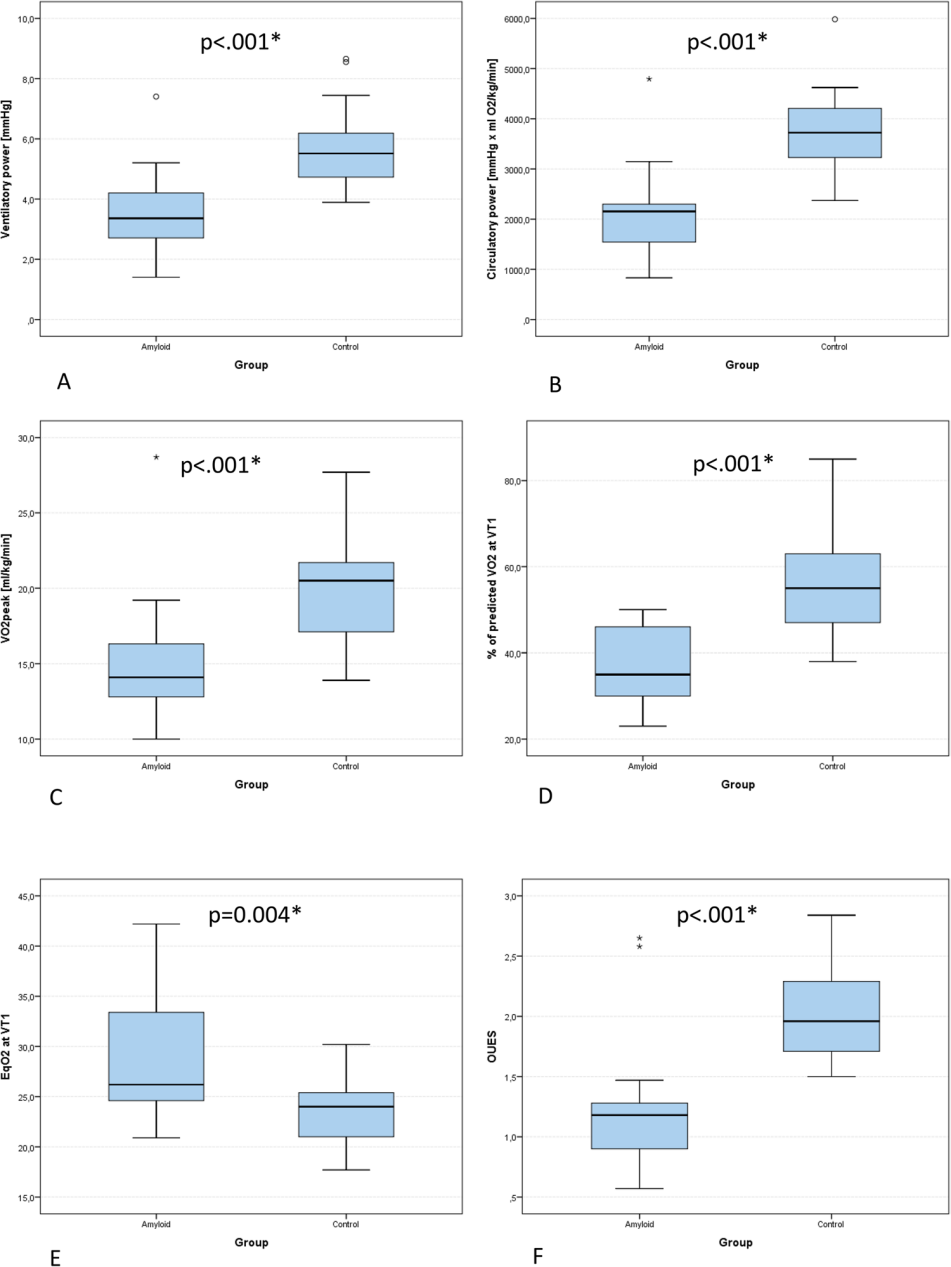

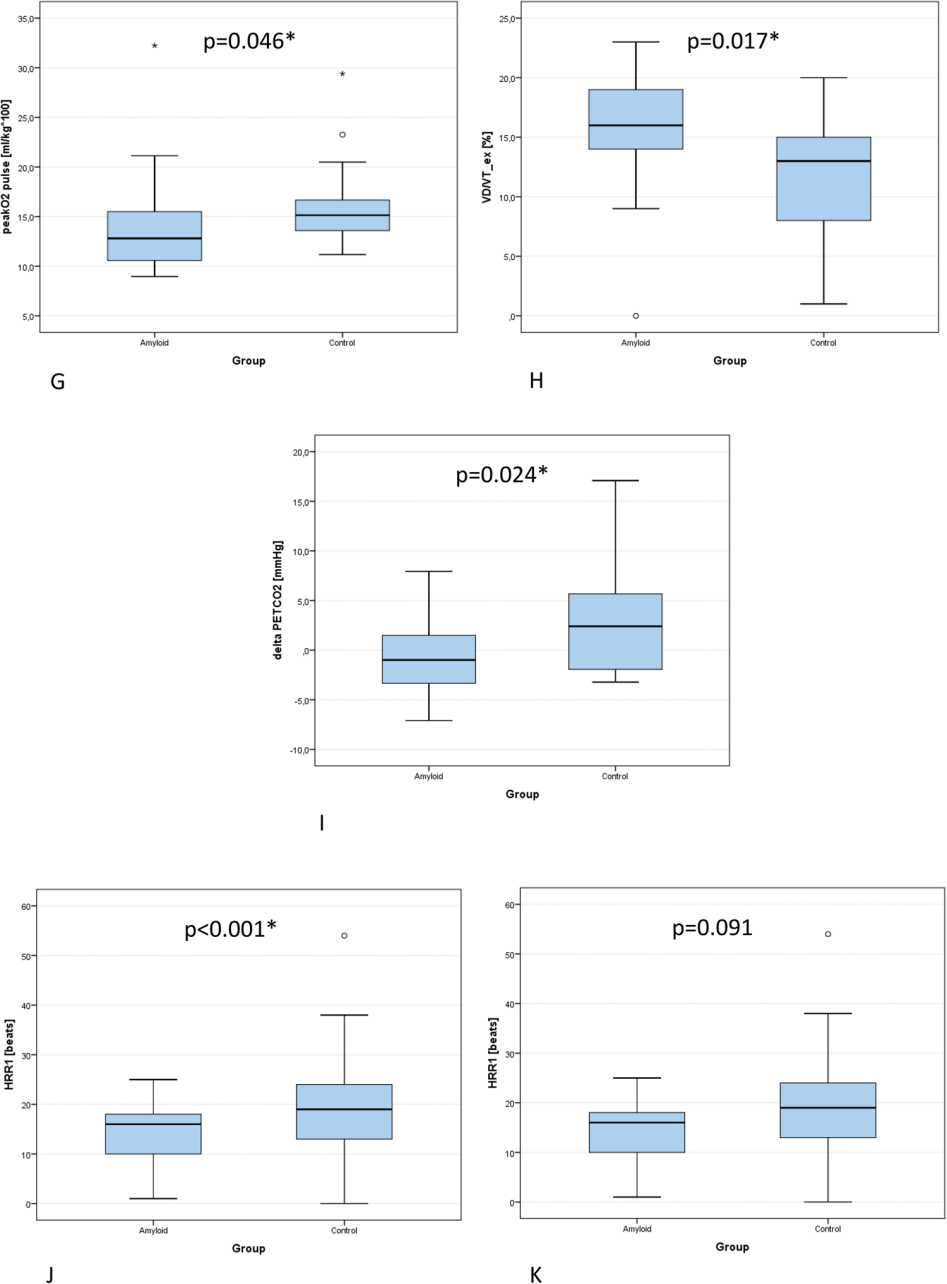
Comparison of cardiopulmonary exercise testing variables in heart failure patients with cardiac amyloidosis (amyloid) and heart failure patients of other aetiologies (control). **A** ventilatory power. **B** circulatory power. **C** VO_2peak_, peak oxygen consumption. **D** Percentage of predicted VO_2_ at the first ventilatory threshold (VT1). **E** Oxygen equivalent at the first ventilatory threshold (EqO_2_ at VT1). **F** Oxygen uptake efficiency slope (OUES). **G** Peak oxygen pulse related to body weight. **H** *V*_D_/*V*_T_: dead space ventilation. **I** Delta PETCO_2_: increase of end tidal CO_2_ from rest to peak exercise. **J** Delta HR: difference between resting and peak heart rate. **K** HRR1: difference of heart rate at peak exercise and heart rate 1 min following exercise termination. Significance is depicted with an asterisk

ΔHR and peak systolic pressure, as well as peak performance and VO_2peak_ were higher in HF (all *p* < 0.001). CA patients displayed more plateauing of O_2_ pulse (*p* = 0.001) and lower O_2_ pulse_max_ (*p* = 0.046, Table 2, Fig. 1). Submaximal CPET variables OUES (*p* < 0.001), VE/VCO_2_ (*p* = 0.004), and EqO_2_ at VT1 (*p* = 0.004) were inferior in CA (Table 2, Fig. 1). CA patients also showed lower values of the compound variables CP and VP (both *p* < 0.001) and lower PETCO_2peak_ (*p* < 0.001) as well as ΔPETCO_2_ (*p* = 0.024), while *V*_D_/*V*_T_ was higher in CA patients (*p* = 0.017, Table 2, Fig. 1). EOV did not differ between groups (*p* = 0.367).

**Table 2:** Comparison of variables of cardiopulmonary exercise testing (CPET) between patients with cardiac amyloidosis (CA) and heart failure controls (HF)

Variables	Min	Q1	Q2	Q3	Max	*p*-value
**HR**_**max**_** (beats/min)**						*p* = 0.005*
CA (*n* = 21)	78.0	96.0	121.0	126.0	155.0	
HFpEF (*n* = 21)	72.0	123.0	129.0	150.0	181.0	
**HR**_**min**_** (beats/min)**						*p* = 0.933
CA (*n* = 21)	53.0	71.0	89.0	96.0	105.0	
HFpEF (*n* = 21)	54.0	75.0	86.0	93.0	101.0	
**ΔHR (beats/min)**						*p* < 0.001*
CA (*n* = 21)	− 3.0	18.0	26.0	42.0	61.0	
HFpEF (*n* = 21)	18.0	41.0	46.0	62.0	80.0	
**HRR1 (beats/min)**						*p* = 0.091
CA (*n* = 21)	1.0	10.0	16.0	18.0	25.0	
HFpEF (*n* = 21)	0.0	13.0	19.0	24.0	54.0	
**RR**_**sysmin**_** (mmHg)**						*p* = 0.048*
CA (*n* = 21)	83.0	105.0	113.0	130.0	172.0	
HFpEF (*n* = 21)	90.0	121.0	129.0	142.0	158.0	
**RR**_**sysmax**_** (mmHg)**						*p* < 0.001*
CA (*n* = 21)	83.0	111.0	141.0	154.0	182.0	
HFpEF (*n* = 21)	118.0	163.0	179.0	206.0	275.0	
**RR**_**diamin**_** (mmHg)**						*p* = 0.031*
CA (*n* = 21)	40.0	60.0	75.0	85.0	95.0	
HFpEF (*n* = 21)	53.0	74.0	83.0	87.0	109.0	
**RR**_**diamax**_** (mmHg)**						*p* = 0.165
CA (*n* = 21)	42.0	64.0	76.0	86.0	107.0	
HFpEF (*n* = 21)	40.0	76.0	85.0	99.0	114.0	
**ΔRR**_**sys**_** (mmHg)**						*p* < 0.001*
CA (*n* = 21)	− 62.0	8.0	22.0	33.0	55.0	
HFpEF (*n* = 21)	− 20.0	37.0	57.0	63.0	133.0	
**ΔRR**_**dia**_** (mmHg)**						*p* = 0.767
CA (*n* = 21)	− 25.0	− 8.0	4.0	9.0	66.0	
HFpEF (*n* = 21)	− 38.0	− 7.0	2.0	10.0	61.0	
**VO**_**2peak**_** (ml/min/kg)**						*p* < 0.001*
CA (*n* = 21)	10.0	13.0	14.0	16.0	29.0	
HFpEF (*n* = 21)	13.9	17.1	20.5	21.7	27.7	
**% of VO**_**2pred**_						*p* < 0.001*
CA (*n* = 21)	33.9	41.0	58.0	63.0	108.0	
HFpEF (*n* = 21)	67.9	81.6	93.4	111.1	117.7	
**% of pred VO**_**2**_** at VT1**						*p* < 0.001*
CA (*n* = 21)	23.0	30.0	35.0	46.0	50.0	
HFpEF (*n* = 21)	38.0	47.0	55.0	63.0	85.0	
***P***_**max**_** (*W*)**						*p* < 0.001*
CA (*n* = 21)	26.0	70.0	87.0	109.0	270.0	
HFpEF (*n* = 21)	79.0	115.0	145.0	154.0	183.0	
**VE (l)**						*p* = 0.041*
CA (*n* = 21)	37.0	47.0	58.0	70.0	146.0	
HFpEF (*n* = 21)	48.0	58.0	76.0	86.0	113.0	
**VO**_**2**_**/W (ml/min/W)**						*p* = 0.007*
CA (*n* = 21)	3.1	7.0	7.0	9.0	11.0	
HFpEF (*n* = 21)	7.1	8.6	9.5	10.4	11.0	
**Peak O**_**2**_** pulse (ml/beat/kg × 100)**						*p* = 0.046*
CA (*n* = 21)	9.0	11.0	13.0	16.0	32.0	
HFpEF (*n* = 21)	11.2	13.6	15.1	16.7	29.4	
**O**_**2**_** pulse**_**min**_** (ml/beat/kg × 100)**						*p* = 0.889
CA (*n* = 21)	3.6	6.0	7.0	10.0	19.0	
HFpEF (*n* = 21)	4.8	6.5	7.4	8.2	18.1	
**ΔO**_**2**_** pulse (ml/beat/kg × 100)**						*p* = 0.172
CA (*n* = 21)	− 0.1	3.0	7.0	9.0	14.0	
HFpEF (*n* = 21)	0.3	5.4	7.7	9.7	17.3	
**EqO**_**2**_** at VT1**						*p* = 0.004*
CA (*n* = 21)	20.9	25.0	26.0	33.0	42.0	
HFpEF (*n* = 21)	17.7	21.0	24.0	25.4	30.2	
**OUES**						*p* < 0.001*
CA (*n* = 21)	0.6	1.0	1.0	1.0	3.0	
HFpEF (*n* = 21)	1.5	1.7	2.0	2.3	2.8	
**VD/VT (%)**						*p* = 0.017*
CA (*n* = 21)	0.0	14.0	16.0	19.0	23.0	
HFpEF (*n* = 21)	1.0	8.0	13.0	15.0	20.0	
**BR FEV**_**1**_** (%)**						*p* = 0.066
CA (*n* = 21)	0.0	16.0	29.0	41.0	59.0	
HFpEF (*n* = 21)	0.0	7.0	22.0	28.0	46.0	
**Circulatory power (ml/kg/min × mmHg)**						*p* < 0.001*
CA (*n* = 21)	830.0	1543.0	2153.0	2298.0	4793.0	
HFpEF (*n* = 21)	2371.2	3227.9	3723.3	4206.5	5983.2	
**Ventilatory power (mmHg)**						*p* < 0.001*
CA (*n* = 21)	1.4	3.0	3.0	4.0	7.0	
HFpEF (*n* = 21)	3.9	4.7	5.5	6.2	8.7	
**VE/VCO**_**2**_						*p* = 0.004*
CA (*n* = 21)	24.6	33.0	42.0	46.0	62.0	
HFpEF (*n* = 21)	19.9	28.8	33.9	35.8	45.9	
**RER**						*p* = 0.116
CA (*n* = 21)	0.90	1.00	1.00	1.00	1.00	
HFpEF (*n* = 21)	0.95	1.02	1.06	1.15	1.24	
**PETCO**_**2rest**_** (mmHg)**						*p* = 0.244
CA (*n* = 21)	20.6	24.0	27.0	30.0	35.0	
HFpEF (*n* = 21)	17.2	17.1	29.6	31.4	33.5	
**PETCO**_**2peak**_** (mmHg)**						*p* < 0.001*
CA (*n* = 21)	19.2	25.0	27.0	28.0	40.0	
HFpEF (*n* = 21)	24.6	29.2	31.3	34.8	41.8	
**ΔPETCO**_**2**_** (mmHg)**						*p* = 0.024*
CA (*n* = 21)	− 7.1	− 3.0	-1.0	1.0	8.0	
HFpEF (*n* = 21)	− 3.2	− 2.0	2.4	5.7	17.1	

Note: Differences of CPET characteristics are calculated with the exact Fisher and Mann–Whitney *U*-tests. Quartiles (*Q*) are depicted for each group. BR FEV_1_: breathing reserve based on resting forced expiratory volume in one second. Circulatory power: Peak oxygen consumption x peak systolic blood pressure. *CPET* cardiopulmonary exercise testing, *HR*_*max*_ maximal heart rate at peak exercise, *HR*_*min*_ resting heart rate. *ΔHR* difference between resting and peak heart rate. *HRR1* heart rate recovery 1 min after exercise termination (difference between peak heart rate and 1 min after exercise termination). *RER* respiratory exchange ratio (VCO_2_/VO_2_), *RR*_*sysmax*_ systolic blood pressure at peak exercise. *RR*_*sysmin*_ systolic blood pressure at rest, *RR*_*diamin*_ diastolic blood pressure at rest, *RR*_*diamax*_ diastolic blood pressure at peak exercise, *ΔRR*_*sys*_ difference between resting and peak systolic pressure, *ΔRR*_*dia*_ difference between resting and peak diastolic pressure, *VO*_*2peak*_ peak oxygen consumption, *% of VO*_*2*_*pred* % of predicted VO_2peak_, *P*_max_ peak performance, *Peak O*_*2*_* pulse* O_2_ pulse at peak exercise related to body weight, *O*_*2*_* pulse*_*min*_ O_2_ pulse at rest related to body weight, *ΔO*_*2*_* pulse* difference between resting and peak O_2_ pulse related to body weight. *EqO*_*2*_* at VT1* oxygen equivalent at the first ventilatory threshold, *OUES* oxygen uptake efficiency slope, *% of pred VO*_*2*_* at VT1* percent of predicted oxygen uptake at the first ventilatory threshold, *VE* respiratory minute volume, *PETCO*_*2peak*_ end tidal carbon dioxide at peak exercise, *PETCO*_*2rest*_ end tidal carbon dioxide at rest, *ΔPETCO*_*2*_ difference between end tidal carbon dioxide at rest and peak exercise, *Ventilatory power* peak systolic pressure / VE/VCO_2_, *VD/VT* dead space ventilation during exercise

Significance was denoted with an asterisk

### CA Patients with Lower Performance Displayed Increased Rehospitalization

**Fig. 2:**
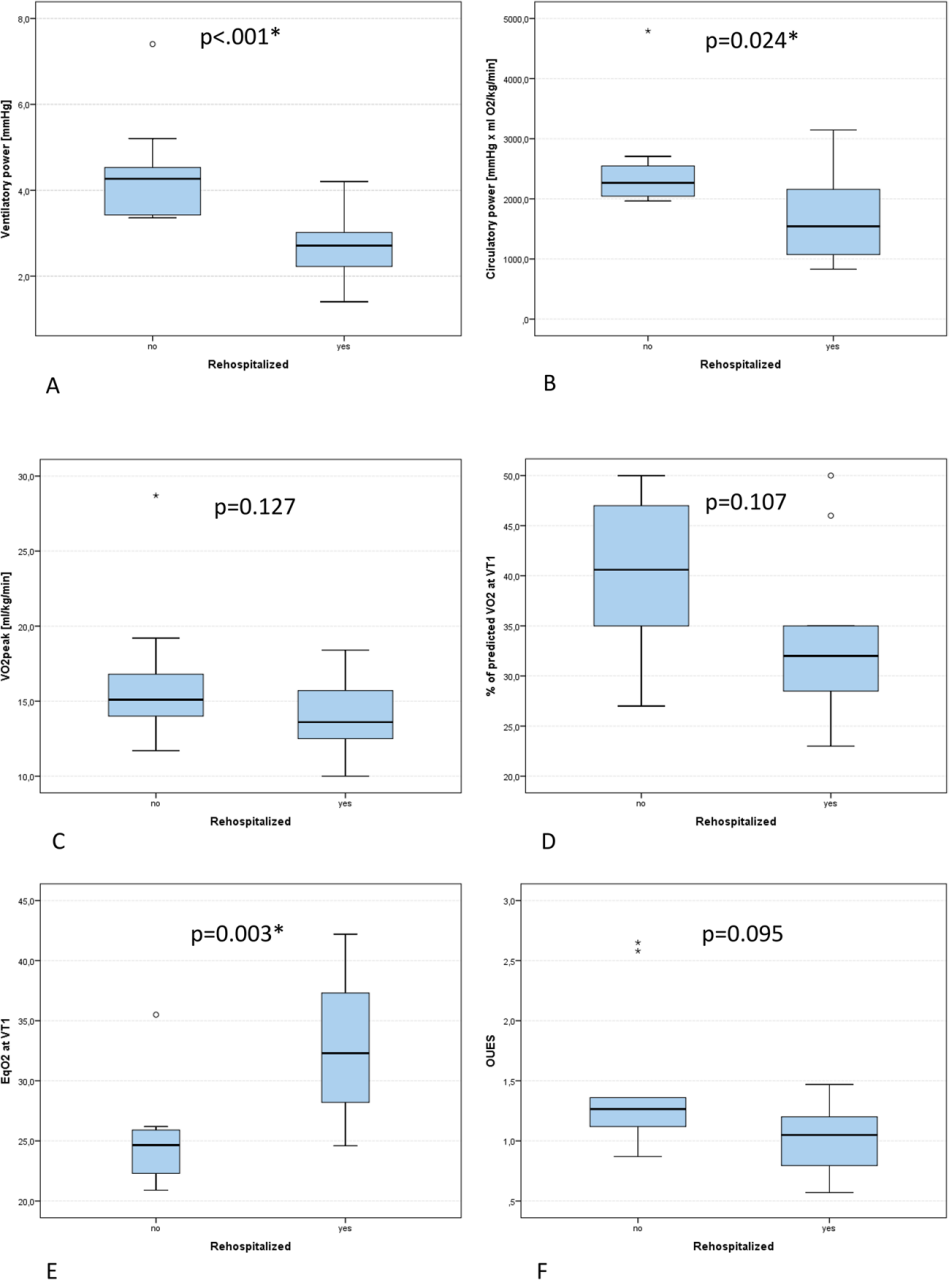

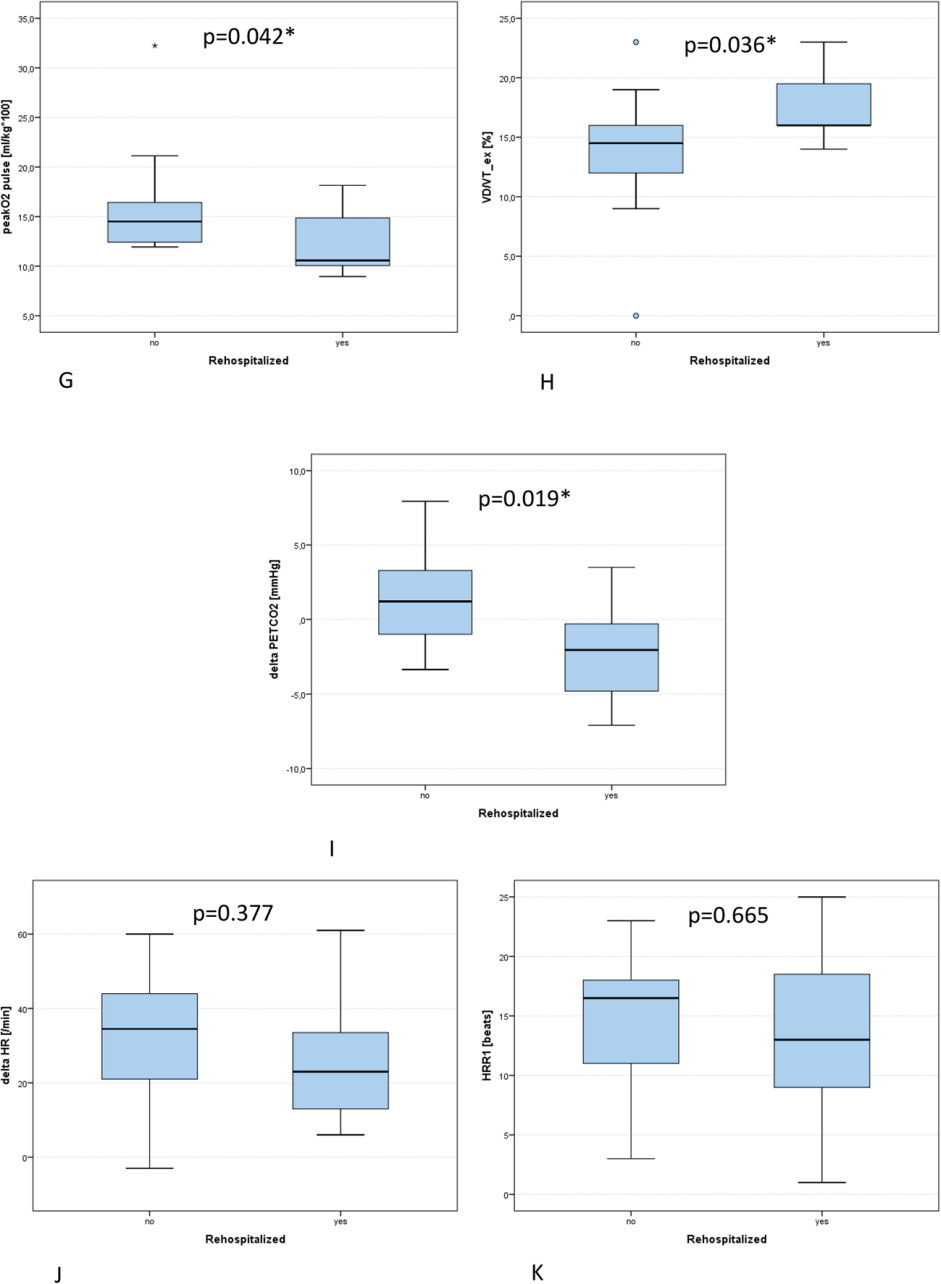
Comparison of cardiopulmonary exercise testing variables in heart failure patients with cardiac amyloidosis with (yes) and without (no) rehospitalization due to heart failure decompensation. **A** Ventilatory power. **B** Circulatory power. **C** VO_2peak_, peak oxygen consumption. **D** Percentage of predicted VO_2_ at the first ventilatory threshold (VT1). **E** Oxygen equivalent at the first ventilatory threshold (EqO_2_ at VT1). **F** Oxygen uptake efficiency slope (OUES). **G** Peak oxygen pulse related to body weight. **H** *V*_D_/*V*_T_: dead space ventilation. **I** Delta PETCO_2_: increase of end tidal CO_2_ from rest to peak exercise. **J** Delta HR: difference between resting and peak heart rate. **K** HRR1: difference of heart rate at peak exercise and heart rate 1 min following exercise termination. Significance is depicted with an asterisk

Rehospitalized CA patients performed worse (*P*_max_ 68.6 ± 25.5 W vs. 116.6 ± 58.0 W, *p* = 0.017), but VO_2peak_ did not differ statistically (13.8 ± 2.7 ml/min/kg vs. 16.5 ± 4.7 ml/min/kg, *p* = 0.127, Fig. 2). The submaximal variables EqO_2_ at VT1 (32.5 ± 6.0 vs. 25.1 ± 4.1, *p* = 0.003), VE/VCO_2_ (46.5 ± 10.1 vs. 36.1 ± 6.8, *p* = 0.019), but not OUES (1.0 ± 0.3 vs. 1.5 ± 0.6, *p* = 0.095) and % of predicted VO_2_ at VT1 (33.5 ± 8.3% vs. 39.6 ± 8.4%, *p* = 0.107), were inferior in hospitalized CA compared to non-hospitalized CA patients (Fig. 2). No differences were displayed in heart rate and blood pressure response to exercise (ΔHR: *p* = 0.377, HRR1: *p* = 0.665, ΔRRsys: *p* = 0.112, ΔRRdia *p* = 0.109). Compound variables VP (2.7 ± 0.8 mmHg vs. 4.4 ± 1.2 mmHg, *p* < 0.001) and CP (1702.4 ± 706.5 ml/kg/min × mmHg vs. 2524.8 ± 833.6 ml/kg/min × mmHg, *p* < 0.001) were lower and ΔPETCO_2_ (− 2.3 ± 3.2 mmHg vs. 1.6 ± 3.8 mmHg, *p* = 0.019) and *V*_D_/*V*_T_ (17.5 ± 2.8% vs. 13.6 ± 6.1%, *p* = 0.036) were inferior in hospitalized CA patients (Fig. 2).

### Correlations Between CPET Parameters

**Fig. 3:**
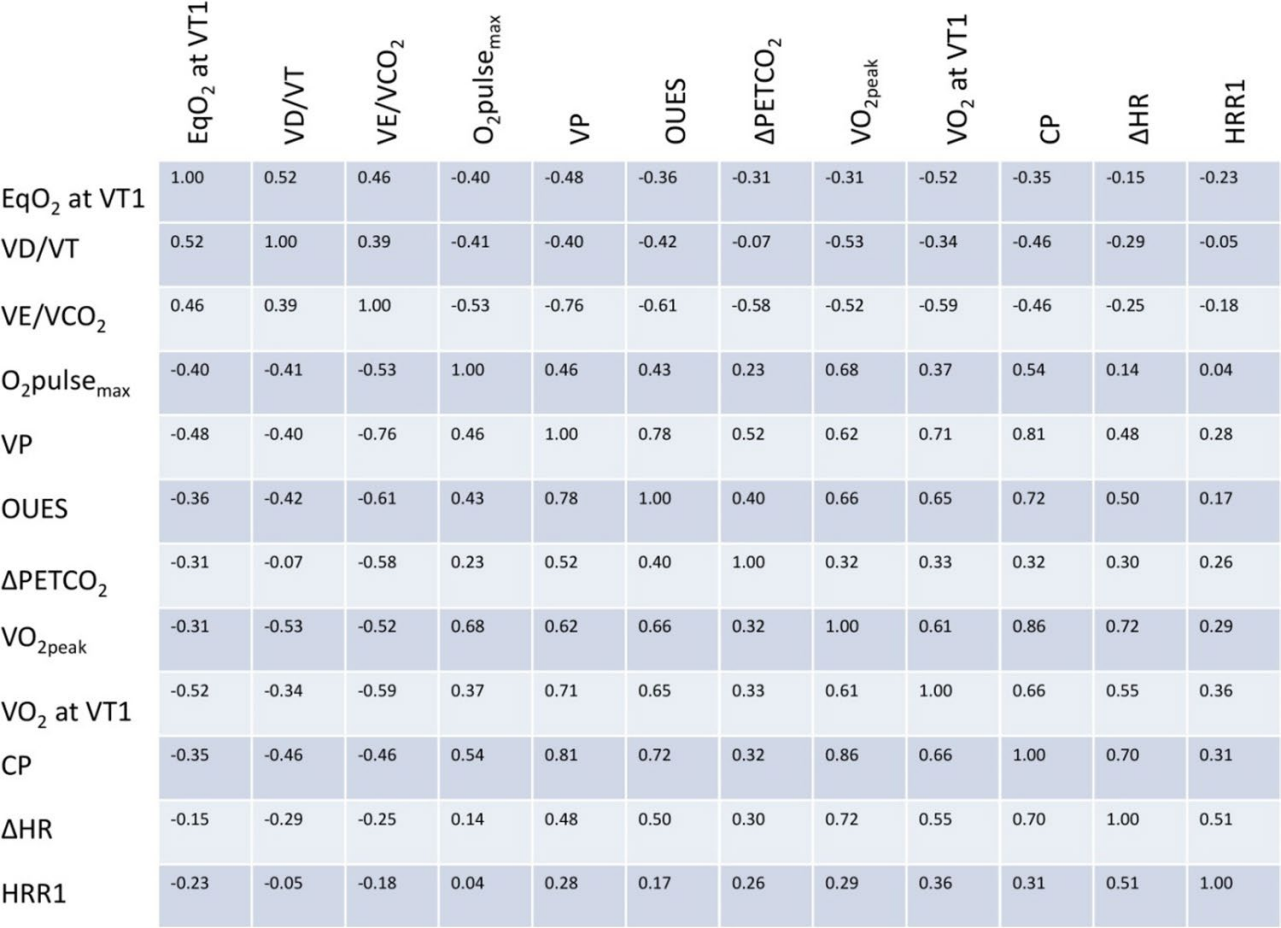
Correlation coefficients between variables of cardiopulmonary exercise testing. EqO_2_ at VT1: oxygen equivalent at the first ventilatory threshold. *V*_D_/*V*_T_: dead space ventilation. VE/VCO_2_: minute ventilation per carbon dioxide production. O_2_pulse_max_: peak oxygen pulse related to body weight. VP: ventilatory power. OUES: oxygen uptake efficiency slope. ΔPETCO_2_: difference of resting and peak end-tidal CO_2_. VO_2peak_: peak oxygen consumption. VO_2_ at VT1: oxygen consumption at the first ventilatory threshold. CP: circulatory power. ΔHR: difference between resting and peak heart rate. HRR1: difference of heart rate at peak exercise and heart rate 1 min following exercise termination. Dark blue represents positive and dark red a negative correlation

Comparisons of CPET variables revealed better correlations for VP with OUES (*r* = 0.78) than VO_2peak_ (*r* = 0.62) and a considerable correlation between VP and CP (*r* = 0.81, Fig. 3). CP was more strongly correlated with VO_2peak_ than OUES (*r* = 0.86 vs. 0.72, Fig. 3). ΔHR was correlated with CP and VO_2peak_ (*r* = 0.70 and 0.72, Fig. 3). The two submaximal variables % of predicted VO_2_ at VT1 and EqO_2_ at VT1 showed a relevant correlation (*r* = 0.71) as well as ΔPETCO_2_ and VE/VCO_2_ (*r* =  − 0.58, Fig. 3).

### Correlations Between CPET Parameters, Echocardiographic Variables, and NTproBNP

**Fig. 4:**
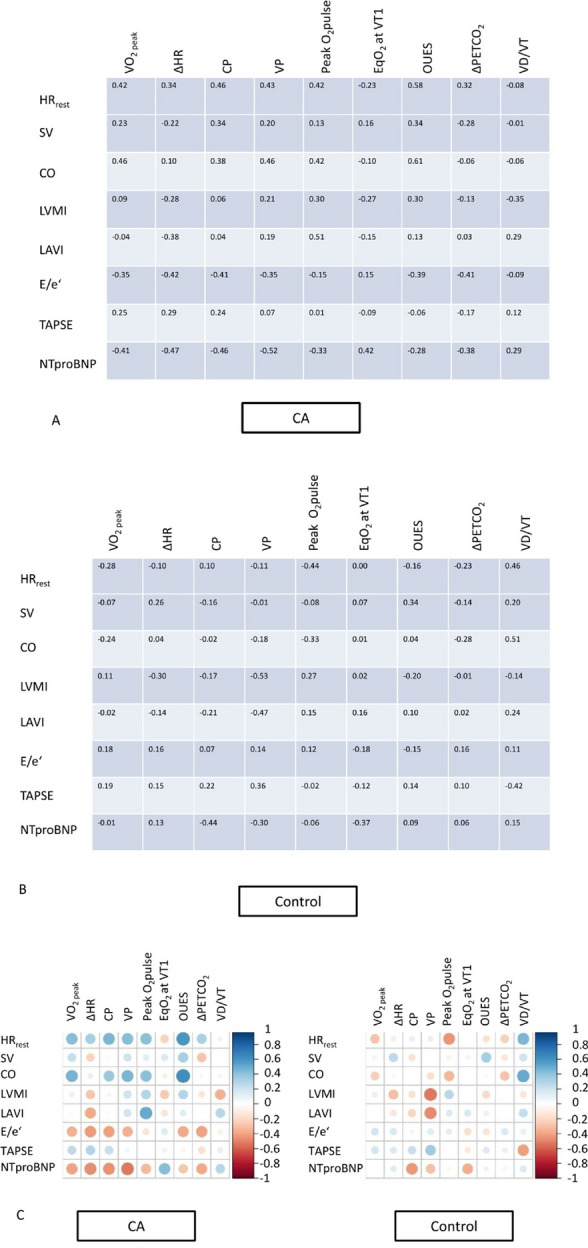
Correlation coefficients between variables of cardiopulmonary exercise testing, echocardiographical variables, and NTproBNP in patients with cardiac amyloidosis (CA, A) and heart failure controls (HF, B). E/e’: mean of lateral and medial E/e’ during resting echocardiography. CO: cardiac output (l/min), calculated by echocardiographic determination of stroke volume times resting heart rate during echocardiography. HR_rest_: resting heart rate during echocardiography (/min). LAVI: left atrial volume index (ml/m^2^). LVMI: left ventricular mass index (g/m.^2^). NTproBNP: N-terminal prohormone of brain natriuretic peptide (pg/ml). SV: stroke volume was calculated by echocardiographic measurement of left ventricular outflow tract diameter and the velocity time integral (l/min). TAPSE: tricuspid annular plane systolic excursion (mm). Circulatory power: peak oxygen consumption x peak systolic blood pressure (ml/kg/min × mmHg). ΔHR: difference between resting and peak heart rate (/min). VO_2peak_: peak oxygen consumption (ml/kg/min). Peak O_2_ pulse: O_2_ pulse at peak exercise related to body weight (ml/beat/kg × 100). EqO_2_ at VT1: oxygen equivalent at the first ventilatory threshold. OUES: oxygen uptake efficiency slope. ΔPETCO_2_: difference between end tidal carbon dioxide at rest and peak exercise (mmHg). Ventilatory power: peak systolic pressure / VE/VCO_2_ (mmHg). VD/VT: dead space ventilation during exercise (%). C: graphical illustration of correlations between CA and HF patients. Correlations range from positive (dark blue) to negative (dark red)

In CA patients, higher HR_rest_, SV, and CO were correlated with better performance indices in CPET (Fig. 4a), while in HF patients, these correlations were less pronounced (Fig. 4b). CA patients demonstrated lower performance with higher NTproBNP and E/e’ (Fig. 4a), while HF patients also showed weaker associations. A graphical comparison of correlations between CA and HF patients is presented in Fig. 4c.

## Discussion

### Overall Findings

Patients with HF due to CA were hospitalized more often than HF controls and were inferior to controls in almost all CPET variables showing lower values of exertional (VO_2peak_, ΔHR, O_2_pulse_max_, ΔPETCO_2_), compound (CP and VP), and non-exertional variables (VE/VCO_2_, OUES, % of predicted VO_2_ at VT1, and EqO_2_ at VT1). Rehospitalization was associated with changes in both exertional (O_2_pulse_max_, V_D_/V_T_, CP, VP, ΔPETCO_2_) and non-exertional variables (VE/VCO_2_, EqO_2_ at VT1), but not with VO_2peak_ and ΔHR in CA patients. Thus, our data supports the notion to implement additional CPET variables to better display exercise limitations apart from the previously shown prognostic value of VO_2peak_ in CA patients [4]. These variables may not only be prognostically relevant but may also help to better understand the pathophysiology of exercise limitations in CA: lower VO_2_ at VT1 and higher EqO_2_ at VT1 in CA patients illustrate that earlier transition from aerobic to anaerobic metabolism is a key determinant of impaired exercise capacity in this vulnerable population. Thus, differentiation between HF in CA and non-CA patients may already be achieved at a submaximal exercise level, which is time-sparing and may even reduce adverse events. This is a novel finding and needs to be further explored in prospective studies.

Our study also illustrates that CA and HF patients display divergent characteristics in echocardiographic and CPET variables, as well as NTproBNP: CA patients demonstrated better performance indices (VO_2peak_, OUES, CP, VP) with higher HR_rest_, SV, and CO but showed lower performance with higher E/e’ and NTproBNP (Fig. 4a, c). Contrary to this, HF patients did not show such an effect. However, HF patients showed higher VP with increased LVMI and LAVI (Fig. 4b, c). This may suggest that in clinical practice, CA patients may benefit from higher resting heart rates. This goes along with previous findings that beta-blockers may be poorly tolerated in amyloidosis, a behavior which can be a marker of unfavorable prognosis [22]. Thus, our data support that beta-blockers should be applied cautiously in this group. Our data may also support the notion that preserved SV and diastolic function (E/e’) at rest are indicators of higher exercise performance in CA patients. These correlations do not seem to be as robust in HF patients. In turn, detection of reduced SV, CO, and higher E/e’ during resting echocardiography in patients with apparently impaired exercise indices (such as VO_2peak_, CP, and VP) should raise suspicion that etiology of heart failure may not be based on hypertension or ischemia but could suggest structural heart disease. Clearly, this hypothesis needs more in-depth analysis.

### Association of the Established Variables VO_2peak_, CP, O_2_pulse_max_, and VE/VCO_2_ on Outcome in CA

Prognosis of CA is limited and has been shown by a retrospective study with wtATTR amyloidosis patients treated with Tafamidis, in which one-third of patients fulfilled the composite primary outcome of mortality, heart transplant, and palliative inotrope initiation after a 1-year follow-up [23•]: low VO_2peak_, CP, and O_2_pulse_max_ were associated with the primary outcome. In addition, it has been shown that O_2_pulse_max_ and VE/VCO_2_ decline over time in CA [24]. The importance of early diagnosis at a better functional state was recently shown by demonstrating that baseline VO_2peak_ > 14 ml/kg/min and VE/VCO_2_ ≤ 34 were associated with a lower risk of death or heart failure rehospitalization before initiation of Tafamidis treatment (*n* = 54, 9 ± 3 months follow-up, mean age 78 ± 6 years, LVEF 52 ± 11) [25]. Although the causality of improvement cannot be attributed to Tafamidis because there was no control group, these findings highlight that CPET contributes to risk stratifying CA and should also trigger exercise trials with CA patients to determine its benefit in addition to Tafamidis. Whether submaximal variables such as EqO_2_ at VT1 can help identify CA patients at risk earlier than exertional variables, such as VO_2peak_, needs to be determined. In our study, VE/VCO_2_ and O_2_pulse_max_ were associated with hospitalization in CA patients, which goes along with the literature [26]. VO_2peak_ did not impact on hospitalization, which may be due to the low case number since values were numerically lower in hospitalized CA. However, CA patients also showed a trend to lower respiratory exchange ratios (RER < 1.05, Table 2), assuming that metabolic exertion was not achieved in this group, and VO_2peak_ may be imprecise [27].

### Impact of CPET Variables to Explain Exercise Limitations in CA

The mechanism of exercise-induced limitations in CA has been demonstrated in a simultaneous study with CPET and right heart catheterization (RHC) showing reduced inotropic reserve during exercise: lower VO_2peak_ was associated with lower SV and peak heart rate, but not with peak pulmonary capillary wedge pressure [28]. Similarly, exercise MRI has unmasked energetic deficits in all four cardiac chambers accounting for transient pulmonary congestion as the pathophysiological substrate of impaired exercise performance [29]. Reduced SV in RHC goes along with CPET findings of reduced O_2_pulse_max_ [3•, 23•], while exercise-induced pulmonary hypertension may be expressed by higher VE/VCO_2_ slopes [3•, 30] and the presence of EOV [31]. Pathophysiologically, myocardial efficiency is hampered, either by the direct toxic effect of amyloid and induction of oxidative stress [32] or structural damage [33]. We confirm these findings in our study by showing lower O_2_pulse_max_ and higher VE/VCO_2_ values in hospitalized CA; numerically, hospitalized CA also showed higher rates of EOV.

CA has also been shown to demonstrate an inadequate heart rate and blood pressure response during exercise [31], which was confirmed in CA compared to HF patients in our study. We also demonstrate that VP, which has scarcely been investigated in CA patients, was associated with hospitalization; thus, this variable should be further studied in CA. Reduced chronotropic reserve, impaired stroke volume development, and peripheral oxygen extraction (lower O_2_pulse_max_) should alert physicians to apply beta-blockers cautiously in CA since this may have deleterious effects on performance and patients’ well-being [34–36].

### Limitations

Our study has several limitations. (1) This was a retrospective, preliminary, and hypothesis-generating trial with a small sample size. (2) We included AL and ATTR amyloidosis in the CA group which, despite proven cardiac involvement, may differ in the pathophysiological response to exercise. (3) Biomarkers and echocardiographic variables showed large variations, which may partly explain the large standard deviations of some CPET variables. (4) As there are significant interactions between CPET variables, as is shown in Fig. 3, no variable should be interpreted on its own but needs to be considered in the context of other components. For instance, it cannot be judged from a single analysis of VE/VCO_2_ whether high values are the result of pre-capillary pulmonary arterial hypertension, insufficient ventilation as a response to diffusion impairment, or postcapillary compromise due to impaired left ventricular compliance during exercise. Larger studies with meticulous echocardiographic and laboratory characterization and stratification for confounding comorbidities are warranted to delineate the chain of exercise impairment in CA. It must be noted that a single CPET variable cannot be used to discriminate the underlying organ dysfunction. As CA and HF have been shown to be limited not only by peak performance induced through the progression of cardiac disease itself but also by reduced muscle capacity [37, 38] and progression of frailty [24], CA itself needs to be regarded as a systemic rather than a mere cardiac disease. Thus, diagnostic workup of CA requires the incorporation of different modalities, such as imaging, CPET, and laboratory markers.

## Conclusion

Exertional and non-exertional CPET performance was inferior in CA compared to HF patients of comparable age and ejection fraction demonstrating more severe exercise limitations. In addition to established exertional variables, such as VO_2peak_, we also illustrate associations of non-exertional CPET variables with hospitalization in CA patients. Correlations between CPET and resting echocardiographic variables seem to differ between CA and HF patients, which emphasizes different properties of exercise response in these patients.

## Data Availability

Data is provided within the manuscript file. Raw data will be made available by the corresponding author on reasonable request.

## Abbreviations

CPET: Cardiopulmonary exercise testing
EOV: Exercise oscillatory ventilation
EqO_2_ at VT1: Oxygen equivalent at the first ventilatory threshold
HFmrEF: Heart failure with mildly reduced ejection fraction
HFpEF: Heart failure with preserved ejection fraction
HFrEF: Heart failure with reduced ejection fraction
NTproBNP: N-terminal prohormone of brain natriuretic peptide
OUES: Oxygen uptake efficiency slope
PETCO_2_: End-tidal CO_2_
VO_2peak_: Peak oxygen consumption
